# Endometrial cancer arising from adenomyosis with unusual manifestation: A case report

**DOI:** 10.1002/ccr3.8353

**Published:** 2023-12-27

**Authors:** Razieh Shahnazari, Shahriar Shirzadi, Sina Karaji, Parisa Mokhles, Mohamad Javad Ghafouri Far, Salome Maghsudlu

**Affiliations:** ^1^ Fellowship in Obstetrics & GYN Imaging, Firoozabadi Clinical Research Development Unit (FACRDU), Department of Radiology, School of Medicine Iran University of Medical Sciences Tehran Iran; ^2^ Department of Radiology Hamedan University of Medical Sciences Hamedan Iran; ^3^ Department of Radiology Iran University of Medical Sciences Tehran Iran; ^4^ Department of Pathology, School of Medicine Iran University of Medical Science Tehran Iran; ^5^ Department of Radiology, School of Medicine, Rasool Akram hospital Iran university of medical sciences Tehran Iran; ^6^ Department of Radiology Tehran University of Medical Sciences Tehran Iran

**Keywords:** Adenomyosis, endometrial cancer, MRI, ultrasonography, uterine cancer

## Abstract

Endometrial Carcinoma Arising from Adenomyosis (EC‐AIA) is an unusual condition, primarily occurring in postmenopausal women. We present a rare case of a 34‐year‐old woman with EC‐AIA, highlighting the diagnostic challenges in distinguishing this malignancy from adenomyosis preoperatively. Conventional imaging methods exhibited limitations, necessitating post‐surgery confirmation through comprehensive examinations. The case emphasizes the need for a multidisciplinary approach for accurate diagnosis and timely management in such uncommon gynecological conditions.

## Graphical abstract

Histological images depict the rarity of Endometrial Carcinoma Arising from Adenomyosis (EC‐AIA) in a 34‐year‐old premenopausal woman. Sections reveal tumoral nests beneath the myometrial layer with distinctive glandular architectures. Diagnostic challenges necessitate post‐surgery confirmation, emphasizing the need for a multidisciplinary approach for accurate diagnosis and timely management in uncommon gynecological conditions.

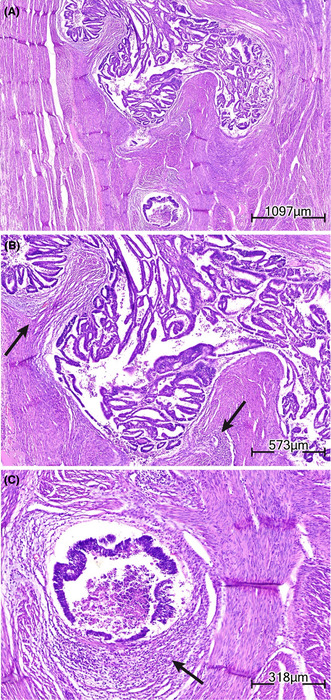

## 1 INTRODUCTION

Endometrial carcinoma is clinically recognized as the malignant alteration of the innermost layer of the uterine lining.^1^ Endometrial cancer accounts for approximately 2.2% of cancers and attributes to 1% of cancer‐related mortalities.^2^ Endometrial carcinoma that develops within adenomyosis, known as EC‐AIA, is an infrequent condition involving the malignant change of the displaced endometrial tissue within the areas affected by adenomyosis.^3^

The diagnostic criteria essential for identifying EC‐AIA include the absence of cancer in the usual endometrium or other pelvic regions. Moreover, the cancer must distinctly emerge from the epithelium of areas affected by adenomyosis, not infiltrating from an alternate source. Lastly, it is pivotal to observe the surrounding endometrial stromal cells encircling the anomalous glands to affirm the diagnosis of adenomyosis.^4^ Based on the limited available data, most EC‐AIA patients are in the postmenopausal phase, with an average age of 58 years. Their predominant clinical presentations encompass abnormal uterine bleeding and pelvic pain.^3^

Adenomyosis is characterized by the existence of abnormal endometrial glands and stroma that are embedded within the muscular wall of the uterus, also known as the myometrium.^5^

Adenomyosis commonly affects pre‐menopausal, multiparous women in their thirties to forties, with a prevalence of 20%–30% among hysterectomy cases.^6^ Its clinical presentation includes symptoms such as abnormal uterine bleeding (AUB), dysmenorrhea, and chronic pelvic pain. However, these manifestations lack pathognomonic features that would enable a definitive differentiation of adenomyosis from other diseases.^7^

Ultrasound is highly accurate for diagnosing gynecological conditions, especially adenomyosis, with a sensitivity of 83.8% and specificity of 63.9%.^8^, ^9^ Despite the significance of MRI in diagnosing uterine issues, clear MRI findings for endometrial cancer arising from adenomyosis are yet to be established.^10^

While the coexistence of endometrial cancer with adenomyosis (EC‐A) is not uncommon, the occurrence of endometrial cancer specifically originating from adenomyosis (EC‐AIA) is extremely rare.^11^ The association between BMI increase and total endometrial cancer risk is significant, along with a correlation between lower risk in parous women.^12^ Evidence linking PCOS to endometrial carcinoma risk is inconclusive. Women with irregular periods or amenorrhea may have a higher risk due to unopposed estrogen secretion.^13^

In this study, we present a rare case of a 34‐year‐old female diagnosed with endometrial cancer arising from adenomyosis (EC‐AIA). The patient exhibited an unusual manifestation of a large lobulated myometrial mass with an evident interruption of the endometrial junction.

## 2 CASE REPORT

A 34‐year‐old woman, a virgin, presented with a three‐year history of abnormal uterine bleeding (AUB) and a body mass index (BMI) of 34. Her medical history revealed a diagnosis of polycystic ovary syndrome (PCOS) without any other known medical conditions or relevant family history. She also underwent hormone therapy for her polycystic ovary condition and received lifestyle modification recommendations. Her menstrual cycles were progressing toward regularity until the adenomyosis was discovered during the follow‐up.

**FIGURE 1:**
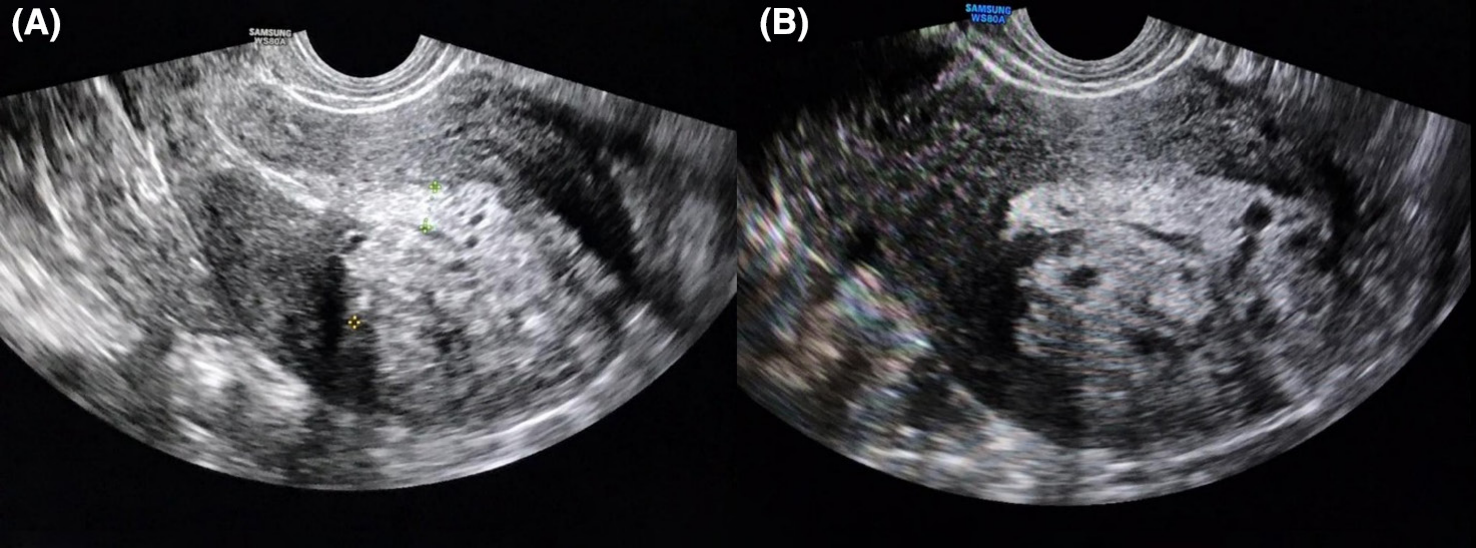
Transrectal sonography reveals complex uterine lesion. (A) Transrectal sonography reveals a globular uterus with asymmetrical myometrial thickening and irregular junctional zones. A lobulated heterogeneous hyperechoic mass‐like lesion, measuring approximately 75 × 57 × 55 mm, is observed at the anterior of the uterus. The lesion displays a few cystic changes and appears to be located at the junctional zone, invading the fundal deep myometrial region. (B) A close‐up view of the hyperechoic mass‐like lesion, showing its lobulated appearance and areas of cystic changes, further highlighting the complexity of the condition.

Initial transabdominal sonography (TAS) revealed a typical presentation of adenomyosis in the uterus, indicated by the indirect findings of a globular shape, asymmetrical myometrial thickening, fan‐shaped shadowing, and an irregular junctional zone in the anterior and posterior endometrium layers (Figure 1). Direct findings included the presence of echogenic subendometrial lines and buds. Notably, a large lobulated hyperechoic mass was found in the fundal region, showing cystic changes and extending from the endometrial to the myometrial region. The origin of this mass was uncertain due to the appearance of nearly well‐defined margins in the outer part of the myometrium and ill‐defined margins surrounding the endometrium.

**FIGURE 2:**
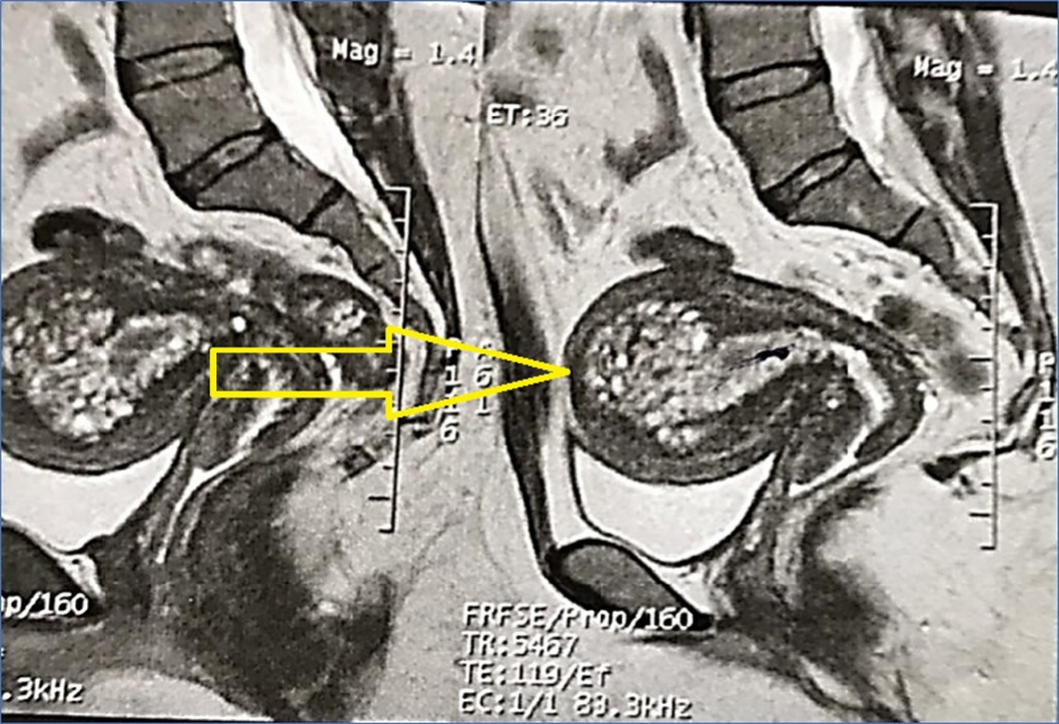
MRI unveils adhesions and uterine mass. MRI with sagittal section reveals an adhesion in the rectovesical space, along with adhesion of both ovaries to the uterus's body, suggesting a possible association with adenomyosis. A high‐signal multicystic, well‐defined mass is observed in the central part of the uterus on T2‐weighted sagittal images, measuring approximately 76 × 53 × 59 mm. The origin of the mass cannot be distinguished as either myometrial or endometrial. It extends near the serosal surface in the left fundal part. The serosal surfaces of the uterus are intact, and there is no evidence supporting the cervical invasion.

Subsequent pelvic MRI revealed diffuse uterine enlargement with a large endometrial‐myometrial mass presenting a multicystic high‐signal lesion in the anterior myometrial body of the uterus, extending to the anterior endometrial layer. The mass's unclear boundaries, along with the patient's severe bleeding, led to further investigations (Figure 2).

Laboratory data revealed normal serum carcinoembryonic antigen (CEA) and carbohydrate antigen (CA) 19–9 values but elevated cancer antigen 125 (CA125). Transrectal ultrasonography (TRS) showed a hyperechoic mass located in the junctional zone, raising suspicions of atypical adenomyosis. A hysteroscopy was conducted, revealing a suspicious polyp‐like lesion; however, its appearance did not distinctly differentiate between primary sources of endometrial cancer.

Based on the high probability of malignancy, the patient underwent total abdominal hysterectomy and bilateral salpingo‐oophorectomy (TAH BSO). Preoperative evaluations were conducted to identify lymph node involvement and metastases. Given the absence of lymph node involvement and metastasis, based on the tumor surgeon's opinion, lymph node dissection was considered unnecessary and was not performed.

**FIGURE 3:**
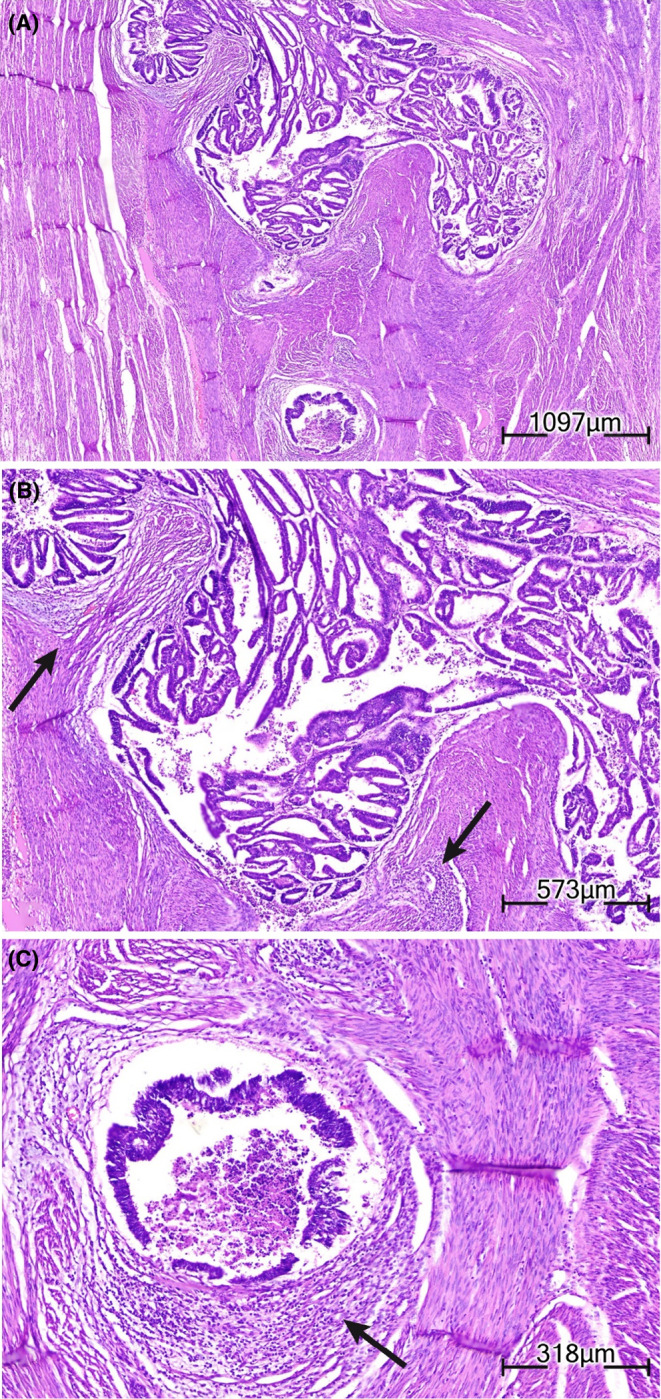
Histological images of endometrial carcinoma arising from adenomyosis. (A) Sections reveal isolated nests of tumoral cells embedded beneath the myometrial layer (×4, H&E). (B) The neoplastic cells are arranged in back to back glands and complex papillary to villoglandular architectures (×10, H&E). (C) Stromal endometrial cell residues are seen focally at the periphery of some nests (black arrows) (×10, H&E).

Post‐operative histologic grading revealed FIGO grade I, more than 50% myometrial invasion, and extensive adenomyosis foci involved by carcinoma. Notably, no uterine serosal involvement, lower uterine segment or cervical stromal involvement, lymphovascular invasion, or regional lymph nodes were identified. Other findings include chronic cervicitis in the cervix and unremarkable right ovary, fallopian tubes, and left ovary (Figure 3). So, after surgery, the criteria set by Colman et al.^4^ for diagnosing EC‐AIA were satisfied based on histopathology. These criteria entail the absence of carcinoma in the usual endometrial locations, identification of carcinoma originating from adenomyotic epithelium, and the presence of surrounding endometrial stromal cells confirming the adenomyosis diagnosis.

Following 25 sessions of radiotherapy, a positron emission tomography (PET) scan was conducted after 2 months to evaluate the possibility of metastasis. The PET scan reported a small pulmonary nodule without fluorodeoxyglucose (FDG) uptake in the posterior segment of the right upper lobe of the right lung, adjacent to the major fissure, which was not considered a metastasis.

## 3 DISCUSSION

Although EC‐AIAs typically occur in postmenopausal ages, here we introduced a case of a 34‐year‐old young woman who was in her fertile years. The most significant challenge in dealing with this case arose in the disease diagnosis phase. Although the results of a meta‐analysis showed that adenomyosis is seen in 22% of endometrial cancer cases (EC‐A), only 1% of cases involve Endometrial Carcinoma Arising in Endometriosis (EC‐AIA).^14^ As observed, while combination of clinical presentation and imaging modalities such as ultrasound and MRI assist in diagnosis, detecting EC‐AIAs often becomes possible post‐surgery and through histological examination.

Based on a recent systematic review of Endometrial Carcinoma Arising in Endometriosis (EC‐AIA) case reports, the postoperative histological examination reveals a distinct pattern. It demonstrates a lower prevalence of the endometrioid histotype and a tendency toward early FIGO stages compared to traditional endometrial carcinoma (EC). Conversely, there is a notably heightened incidence of abnormal p53 expression and a greater requirement for adjuvant treatment in EC‐AIA cases.^3^ In the case we presented, we observed FIGO Stage IB, indicating that the tumor had spread to one‐half or more of the myometrium.

In our case, the radiological findings align with direct Morphological Uterus Sonographic Assessment (MUSA) features for diagnosing adenomyosis, including the presence of myometrial cysts, hyperechoic islands, and hyperechoic sub‐endometrial lines or buds, along with four out of five indirect MUSA features, namely asymmetrical myometrial thickening, fan‐shaped shadowing, irregular junctional zone (JZ), and interrupted JZ.^9^ Consequently, despite its unusual appearance, our case was in line with the MUSA adenomyosis criteria.

Several differential diagnoses of adenomyosis include physiological myometrial contraction, pregnant uterus, and primary and secondary malignancies.^15^ Additionally, pseudo‐widening of the endometrium can obscure the endometrial junction. However, further radiological examination ruled out physiological myometrial contraction and pseudo‐widening of the endometrium.

In malignancies, MR imaging often shows restricted diffusion, indicating increased cellularity due to the restricted movement of water in highly cellular tumor tissues.^16^ Although our case showed negative diffusion restriction, not suggestive of cancer, the unclear boundaries between endometrial and myometrial regions, the unknown origin of the mass, and the presence of persistent AUB prompted specialists to consider the possibility of malignancy alongside adenomyosis.

Several co‐factors in our case contributed to the increased probability of malignancy, including high BMI, nulliparity, and polycystic ovary syndrome (PCOS), although the age was not typical for endometrial cancer.^12^, ^13^

Transrectal ultrasonography (TRS) and hysterectomy confirmed the high likelihood of malignancy, revealing a mass with an irregular appearance. As a result, radiologists proposed the following possibilities based on the imaging review: low‐grade endometrial cancer penetrating the deep endometrium, atypical adenomyosis with polypoid extension to the endometrium, and atypical myometrial masses such as low‐grade endometrial stromal sarcoma.

Given the critical consideration of the patient's age and the potential impact of delaying the removal of any possible cancer on prognosis, the final decision was to perform a total abdominal hysterectomy and bilateral salpingo‐oophorectomy (TAH BSO) with the patient's informed consent. The pathology report from full‐thickness specimens confirmed endometrial cancer arising from adenomyosis.

## 4 CONCLUSIONS

This case report underscores the diagnostic complexity of endometrial cancer arising from uterine adenomyosis. The combination of radiologic modalities and pathology examination proved essential for arriving at an accurate diagnosis. The presented case exemplifies the challenges in identifying and differentiating this rare manifestation, emphasizing the need for meticulous evaluation and interdisciplinary collaboration. Timely and precise management remains crucial in optimizing patient outcomes.

## AUTHOR CONTRIBUTIONS

**Razieh Shahnazari:** Conceptualization; investigation; project administration; resources. **Shahriar Shirzadi:** Data curation; writing – original draft. **Sina Karaji:** Validation; visualization; writing – review and editing. **Parisa Mokhles:** Writing – review and editing. **Mohamad Javad Ghafouri Far:** Formal analysis; investigation. **Salome Maghsudlu:** Investigation.

## FUNDING INFORMATION

There is no funding for this article – This research was conducted without the financial support of any specific funding agency or organization. The authors independently carried out the study without any external funding sources, highlighting the impartiality and independence of the research findings.

## CONFLICT OF INTEREST

The authors declare that they have no financial and non‐financial competing interests. All authors involved in the research declare that they have no financial relationships or other interests that could influence the content or interpretation of the findings presented in this article.

## ETHICS STATEMENT

Informed consent was obtained from the participant, who was provided with detailed information about the study objectives, procedures, potential risks, and benefits. The participant voluntarily agreed to participate by signing a written consent form. Confidentiality and privacy of the participant's personal information were strictly maintained throughout the study.

## CONSENT

Written consent for publication was obtained from the participant. The participant was informed about the use of their data for research purposes and publication. They were assured that their identity would be protected, and their data would be anonymized in any publication. The participant had the option to withdraw their consent at any time before publication without any consequences.

## Data Availability

The MRI images and ultrasonography data used in this article are available from the corresponding author upon reasonable request. Researchers interested in accessing the data may contact the corresponding author for further information on how to obtain the datasets used in this study.

## References

[1] Makker V , Mackay H , Ray‐Coquard I , et al. Endometrial cancer. Nat Rev Dis Primers. 2021;7(1):88. doi:10.1038/S41572-021-00324-8 34887451 PMC9421940

[2] Sung H , Ferlay J , Siegel RL , et al. Global cancer statistics 2020: GLOBOCAN estimates of incidence and mortality worldwide for 36 cancers in 185 countries. CA Cancer J Clin. 2021;71(3):209‐249. doi:10.3322/caac.21660 33538338

[3] Raffone A , Raimondo D , Maletta M , et al. Endometrial Cancer Arising in Adenomyosis (EC‐AIA): a systematic review. Cancers (Basel). 2023;15(4):1142. doi:10.3390/cancers15041142 36831484 PMC9953860

[4] Colman HI , Rosenthal AH . Carcinoma developing in areas of adenomyosis. Obstetrics & Gynecology. 1959;14(3):342‐348. 13811320

[5] Habiba M , Benagiano G . What is Adenomyosis? In: Oral E , ed. Endometriosis and Adenomyosis: Global Perspectives Across the lifespan. Springer International Publishing; 2022:399‐410.

[6] Gunther R , Walker C . Adenomyosis. StatPearls. StatPearls Publishing; 2023. 30969690

[7] Peric H , Fraser IS . The symptomatology of adenomyosis. Best Pract Res Clin Obstet Gynaecol. 2006;20(4):547‐555. doi:10.1016/j.bpobgyn.2006.01.006 16515888

[8] Van den Bosch T , de Bruijn AM , de Leeuw RA , et al. Sonographic classification and reporting system for diagnosing adenomyosis. Ultrasound Obstet Gynecol. 2019;53(5):576‐582. doi:10.1002/uog.19096 29790217

[9] Harmsen MJ , Van den Bosch T , de Leeuw RA , et al. Consensus on revised definitions of morphological uterus sonographic assessment (MUSA) features of adenomyosis: results of modified Delphi procedure. Ultrasound Obstet Gynecol. 2022;60(1):118‐131. doi:10.1002/uog.24786 34587658 PMC9328356

[10] Izumi Y , Yamamoto T , Matsunaga N , et al. Endometrial cancer arising from adenomyosis: case report and literature review of MRI findings. Radiol Case Rep. 2020;15(4):427‐430. doi:10.1016/j.radcr.2020.01.025 32099587 PMC7031131

[11] Machida H , Maeda M , Cahoon SS , et al. Endometrial cancer arising in adenomyosis versus endometrial cancer coexisting with adenomyosis: are these two different entities? Arch Gynecol Obstet. 2017;295(6):1459‐1468. doi:10.1007/s00404-017-4375-z 28444512 PMC7523234

[12] Raglan O , Kalliala I , Markozannes G , et al. Risk factors for endometrial cancer: an umbrella review of the literature. Int J Cancer. 2019;145(7):1719‐1730. doi:10.1002/ijc.31961 30387875

[13] Hardiman P , Pillay OC , Atiomo W . Polycystic ovary syndrome and endometrial carcinoma. Lancet. 2003;361(9371):1810‐1812. doi:10.1016/s0140-6736(03)13409-5 12781553

[14] Raffone A , Seracchioli R , Raimondo D , et al. Prevalence of adenomyosis in endometrial cancer patients: a systematic review and meta‐analysis. Arch Gynecol Obstet. 2021;303(1):47‐53. doi:10.1007/s00404-020-05840-8 33098006 PMC7854401

[15] Takeuchi M , Matsuzaki K . Adenomyosis: usual and unusual imaging manifestations, pitfalls, and problem‐solving MR imaging techniques. Radiographics. 2011;31(1):99‐115. doi:10.1148/rg.311105110 21257936

[16] Jha RC , Zanello PA , Ascher SM , Rajan S . Diffusion‐weighted imaging (DWI) of adenomyosis and fibroids of the uterus. Abdom Imaging. 2014;39(3):562‐569. doi:10.1007/s00261-014-0095-z 24531353

